# Old drug, new tricks: the utility of metformin in infection and vaccination responses to influenza and SARS-CoV-2 in older adults

**DOI:** 10.3389/fragi.2023.1272336

**Published:** 2023-10-11

**Authors:** Dominique E. Martin, Andreia N. Cadar, Jenna M. Bartley

**Affiliations:** UConn Center on Aging, Department of Immunology, University of Connecticut School of Medicine, Farmington, CT, United States

**Keywords:** aging, metformin, influenza, COVID-19, geroscience

## Abstract

In the face of global pathogens such as influenza (flu) and severe acute respiratory syndrome coronavirus 2 (SARS-CoV-2), strategies beyond standard vaccines and virus-specific treatments are critically needed for older populations who are more susceptible to severe disease and death from these infections due to age-related immune dysregulation. Thus, complimentary therapeutics are needed to address the increased risk of complications and death in older adults. Metformin, an FDA approved diabetes drug, is an attractive therapeutic candidate to improve immune defenses and resilience in older adults facing viral challenge. Metformin is already a candidate anti-aging drug, but its benefits have potential to span beyond this and improve specific immune responses. Metformin can target multiple aging hallmarks as well as directly impact innate and adaptive immune cell subsets. Both retrospective and prospective studies have demonstrated metformin’s efficacy in improving outcomes after SARS-CoV-2 or flu infections. Moreover, evidence from clinical trials has also suggested that metformin treatment can improve vaccination responses. In totality, these findings suggest that metformin can improve age-related declines in immunological resilience. Strategies to improve outcomes after infection or improve vaccine-induced protection are invaluable for older adults. Moreover, the ability to repurpose an already FDA approved drug has significant advantages in terms of necessary time and resources. Thus, metformin has great potential as a therapeutic to improve age-related immune dysregulation during flu and SARS-CoV-2 infections and should be further explored to confirm its ability to improve overall immunological resilience in older adults.

## Introduction

The goal of geroscience research is to target the biological drivers of aging to simultaneously decrease risk for multiple age-related diseases and improve resilience in older adults (Justice et al., 2021). Aging underlies many chronic conditions such as diabetes, cancer, Alzheimer’s disease, dementia, chronic obstructive pulmonary disease (COPD), osteoarthritis, and others (Jaul and Barron, 2017; Rea et al., 2018). Further, older adults face immune declines with aging, making them more susceptible to illness and chronic conditions (Nikolich-Zugich, 2018). Therefore, therapeutics that can target the biological drivers of aging are of great interest and have the potential to address multiple conditions at once and improve overall resilience and healthspan in older adults.

In the face of global pathogens such as influenza (flu) and severe acute respiratory syndrome coronavirus 2 (SARS-CoV-2), additional strategies beyond standard vaccines and virus-specific treatments are critically needed for older populations. It is known that older adults are more susceptible to severe disease and death with flu and SARS-CoV-2 infections. Thus, complimentary therapeutics are needed to address the increased risk of complications and death in older adults. Theoretically, therapeutics that target aging biology would also improve immune responses to both vaccination and overall health outcomes following pathogen exposure. Drugs that could either reduce recovery time, chronic pathology, or frailty and disability following infection would be invaluable when combatting pathogens in older adults. Further, treatments that could improve immunological memory formation following vaccination could potentially prevent severe infections from vaccine-preventable illnesses, such as flu and SARS-CoV-2, in older adults.

## Age-related declines in the immune system

One of the most recognized consequences of aging is a decline in immune function and ability to fight infection (Montecino-Rodriguez et al., 2013; Pinti et al., 2016; Weyand and Goronzy, 2016; Sadighi Akha, 2018). While respiratory illnesses such as flu and SARS-CoV-2 impact people of all ages, older adults are at a disproportionately high risk for severe disease, complications, and death. This has been observed with flu for decades and has been further highlighted during the COVID-19 pandemic. In fact, in the United States, approximately 75% of COVID-19 deaths occurred in adults over the age of 65 (CDC and Centers for Disease Control and Prevention, 2023). Additionally, older adults account for 80% of hospitalizations and have a 23-fold greater risk of death than those under 65 (Mueller et al., 2020). These negative consequences of COVID-19 in older populations are paralleled with flu infections. Importantly, a study of global flu mortality reported that 67% of deaths associated with this disease were among people 65 years and older (Paget et al., 2019). On top of this, older adults face increased risk of hospitalization and complications after contracting one of these viral illnesses (Czaja et al., 2019; Mueller et al., 2020). Complications include increased risk of secondary pneumonia, altered mental status, heightened risk of cardiovascular events, increased risk of fracture or fall injury during the first year after discharge, difficulties conducting activities of daily living, and others (Thompson et al., 2004; Czaja et al., 2019; Mueller et al., 2020; Axelsson et al., 2022). Indeed, both SARS-CoV-2 and flu are leading killers of older adults due to age-related declines in immune responses to infectious diseases and reduced vaccine-induced protection. However, the increased risk of complications post-infection present other challenges older adults face when contracting viral illness. Immune changes with aging impact the ability of older adults to both fight infection and recover. Therefore, finding strategies that would help older adults better fight infection or boost responses to vaccination is a critical public health initiative.

There are distinct changes to both the innate and adaptive arms of the immune system that results in phenotypic and functional changes in immune responses [Reviewed in depth at Solana et al. (2006), Shaw et al. (2010), Nikolich-Zugich (2018), and Sadighi Akha (2018)]. More specifically, cells of the innate immune system such as neutrophils, macrophages, dendritic cells, and natural killer (NK) cells face altered effector functions including impaired phagocytosis, antigen presentation, and overall cytotoxicity (Solana et al., 2006). Further, macrophage dysregulation is thought to be a primary driver of inflammaging (Oishi and Manabe, 2016; Yarbro et al., 2020), defined as the increased chronic, sterile low-grade inflammatory status observed with age (Franceschi et al., 2000). Inflammaging is a substantial risk factor for morbidity and mortality in older adults and further impairs immune responses due to microenvironment changes (Franceschi et al., 2000).

The adaptive immune system is similarly burdened with age. With aging, there are reduced naïve populations of T cells, decreased TCR diversity, reduced TCR signaling, as well as compromised effector functions (Nikolich-Zugich, 2018). There are also increased memory-like precursor cells known as virtual memory T cells (Nikolich-Zugich, 2018). CD4 T cells have altered subset differentiation patterns and effector function with age, and CD8 T cells have impaired cytotoxicity and poor differentiation into effector cells (Nikolich-Zugich, 2018). Overall, these impact primary response to pathogens, memory cell formation, and recall responses with aging (Nikolich-Zugich, 2018). Further, B cells also exhibit reduced numbers of naïve populations, and impaired functionality with reduced quantity and quality of antibodies (Nikolich-Zugich, 2018). In totality, immune dysregulation with aging impairs immune responses to infection and vaccination, putting older adults at higher risk for severe infection and death due to infectious diseases. Therefore, therapeutics that can target aging biology as well as immune declines are of great interest when combatting acute and chronic diseases.

## Metformin mechanisms and aging biology

Metformin (1,1-dimethylbiguanide) is a first-line treatment for type 2 diabetes (T2D) (LaMoia and Shulman, 2021). Foundational studies in the 1980s and 1990s showed strong evidence of metformin’s efficacy in reducing hyperglycemia in patients with T2D with minimal side effects (Hermann, 1979; Schafer, 1983; Jackson et al., 1987). Later studies confirmed that metformin effectively improved peripheral insulin sensitivity in patients with T2D (Gin et al., 1985; Pedersen et al., 1989) and it was FDA approved in 1994 and used widely since then. However, the exact mechanism of action for metformin is not completely understood. Several studies suggest that metformin works through multiple modes of action. One of the primary mechanisms for antidiabetic effects is through the inhibition of hepatic glucose production, which leads to a decrease in blood glucose levels (Rena et al., 2017). Metformin also has secondary effects on skeletal muscle and gut microbiota (LaMoia and Shulman, 2021) to improve glucose homeostasis. Metformin additionally activates AMP-activated protein kinase (AMPK), which plays a crucial role in regulating glucose and lipid metabolism (Zhou et al., 2001) and inhibits mammalian target of rapamycin (mTOR) and Complex I of the mitochondrial respiratory chain (Rena et al., 2017).

Importantly, metformin targets the molecular and cellular drivers of aging and has the potential to exert positive effects on multiple aging systems (Kulkarni et al., 2020; Justice et al., 2021). Of note, metformin was shown to extend the lifespan of *C. elegans* (Chen et al., 2017) and mice (Martin-Montalvo et al., 2013) as well as reduce all-cause mortality in humans (Campbell et al., 2017). More specifically, metformin can attenuate multiple hallmarks of aging through it pleotropic effects (Lopez-Otin et al., 2013; Kulkarni et al., 2020). Dysregulated metabolism with aging is hallmarked by changes such as deregulated nutrient sensing, mitochondrial dysfunction, and inflammation (Lopez-Otin et al., 2013). Metformin has beneficial effects on energy metabolism in diabetics and prediabetics, and likely non-diabetic older adults. As an established AMPK activator and known modulator of mTOR signaling (Kalender et al., 2010; Onken and Driscoll, 2010; Rena et al., 2017), the role of metformin in modulating nutrient-sensing pathways has been thoroughly investigated. For example, metformin has been shown to improve metabolism by modulating the functional capacity of mitochondria, which are known to become dysfunctional with age (Su et al., 2016; Srivastava, 2017). Metformin improves mitochondrial biogenesis via PGC-1α and lowers oxidative stress by inhibiting Mitochondrial Complex I (Fontaine, 2018). Further, metformin can target other aging hallmarks such as altered intracellular communication and inflammation. Metformin has direct anti-inflammatory action via inhibition of nuclear factor κB (NFκB) via AMPK-dependent and independent pathways (Saisho, 2015) and has been shown to suppress pro-inflammatory cytokines (Zhou et al., 2016; Giusti et al., 2022). Studies have also shown that metformin can improve metabolic parameters such as hyperglycemia, insulin resistance, and atherogenic dyslipidemia (Lee et al., 2022) that contribute to inflammatory processes. In addition, metformin can modulate the gut microbiome (Cabreiro et al., 2013; Sun et al., 2018; Pryor et al., 2019), further reducing inflammation and improving metabolism. On top of this, metformin can affect cellular senescence and has been shown to decrease senescent cell burden and downregulate the senescent associated secretory phenotype (SASP) which is known to contribute to age associated inflammation (Moiseeva et al., 2013; Noren Hooten et al., 2016; Kuang et al., 2020).

Metformin targets multiple aging hallmarks, even those less associated with energy metabolism and inflammation. Epigenetic alterations are characteristic of biological aging and include changes in histone modifications, DNA methylation, and chromatin (Sen et al., 2016). Metformin targets these age-related changes and has been shown to regulate transcriptional activity via histone modifications, DNA methylation, and miRNAs (Bridgeman et al., 2018). Telomere attrition, another hallmark of aging, is associated with biological aging, frailty, age-related morbidities, and mortality (Armanios et al., 2009; Araujo Carvalho et al., 2019; Whittemore et al., 2019). In multiple studies, metformin has been shown to reduce telomere shortening in diabetic individuals (Rosa et al., 2018; Liu et al., 2019). Loss of proteostasis is another hallmark that metformin can target. Mechanisms such as protein synthesis, protein folding, autophagy-mediated protein degradation, and maintenance of conformational stability can all become impaired with aging leading to protein abundance (Hipp et al., 2019). Metformin targets these protein level changes and has been shown to rescue protein misfolding as well as augment autophagy (Shi et al., 2012; Kaushik and Cuervo, 2015; Lu et al., 2016; Athanasiou et al., 2017). Aging is also characterized by deficits in tissue regenerative capacities as well as impairments to stem and progenitor cells which are essential for maintaining homeostasis (Lopez-Otin et al., 2013; Schultz and Sinclair, 2016; Ahmed et al., 2017). Metformin additionally targets these cell types by inducing stem cell rejuvenation and delaying stem cell aging (Na et al., 2013; Fatt et al., 2015; Neumann et al., 2019).

Clearly, metformin can act on multiple hallmarks of aging through various mechanisms, making it a candidate therapeutic to target multiple aspects of aging biology (Kulkarni et al., 2020). Additionally, metformin lowers the risks of age-related conditions such as cardiovascular disease and cancer as well as reduces all-cause mortality in diabetic patients (Han et al., 2019; Saraei et al., 2019). In several animal models, metformin can extend lifespan and some aspects of healthspan (Mohammed et al., 2021; Chen et al., 2022). Importantly, the benefits of metformin are seen without major risk of hypoglycemia. Moreover, metformin has an excellent safety profile with over 5 decades of clinical use, is generally well tolerated in large and diverse populations of older adults, and is a generic and relatively inexpensive medication (Justice et al., 2021). Due to its safety, availability, affordability, and utility in treating age-related conditions, metformin is a leading therapeutic candidate for combatting many of the health concerns older populations face.

## Metformin and the immune system

Along with targeting these biological drivers of aging, metformin directly impacts immune function in many different contexts. Immune decline with age makes older adults more susceptible to disease and chronic conditions. Immune aging is driven by systemic changes in inflammation as well as alterations to immune cell subsets as described above. Metformin’s effect on inflammation has been well studied and this drug has been shown to reduce cytokine levels in multiple contexts (Saisho, 2015). Interestingly, the anti-inflammatory properties of metformin were demonstrated irrespective of diabetes status in a cohort of adult participants (Cameron et al., 2016). Additionally, it is hypothesized that one mechanism by which metformin may alleviate the severity of COVID-19 is through modulation of the cytokine storm (Zangiabadian et al., 2021). IL-6 is one of the main cytokines involved in COVID-19 pathogenesis (Bain et al., 2021). Indeed, metformin has been shown to reduce the secretion of IL-6 and IL-1β by macrophages primed with the COVID-19 spike protein (Xian et al., 2021). On top of this, inflammation is a critical driver of age-related dysfunction including immune decline. Therefore, metformin’s ability to target excess inflammation is extremely promising in improving immune regulation and responses in older adults.

Further research has demonstrated that metformin has specific immunomodulatory effects that are context and cell specific. For example, metformin is known to impact monocytes, macrophages, and other cells of the mononuclear phagocyte system (MPS, which are part of the front-line immune defense against infections). Broadly, macrophages can be divided into two phenotypes, M1 which is pro-inflammatory and M2 which is anti-inflammatory. Multiple studies have demonstrated that metformin can stimulate the M1 to M2 phenotype switch, promoting an anti-inflammatory response. In one study, metformin was able to reduce tumor progression and angiogenesis by promoting the M2 switch (Wang et al., 2019). Another study found that in obese mice, metformin was able to reduce the amount of monocyte chemoattractant protein 1 (MCP-1) and the number of M1 macrophages in adipose tissue while also increasing the M2:M1 ratio (Jing et al., 2018). Further research confirmed that metformin can promote the M1 to M2 switch in other disease contexts such as hyperlipidemia, wound healing, and ischemic stroke (Jin et al., 2014; Qing et al., 2019; Seneviratne et al., 2021). All together, these studies demonstrate that metformin can reduce the severity of inflammation through its modulation of innate immune cells such as monocytes and macrophages.

Metformin also impacts T cells and adaptive immune responses. For example, metformin has been shown to ameliorate T cell mediated inflammation by inhibiting T-cell trafficking and activation, inducing regulatory T cell (Treg) polarization, and inhibiting signal transducer and activator of transcription (STAT) 3 signaling (Nyambuya et al., 2020). More specific to aging, *in vitro* treatment with metformin enhanced autophagy and normalized mitochondrial function in aged CD4 T cells, ameliorating the Th17 inflammaging profile (Bharath et al., 2020). Other reports have demonstrated that metformin reduces the number of pro-inflammatory Th17 cells and increases Tregs, which are essential to regulating or suppressing other immune cells (Son et al., 2014; Sun et al., 2016; Duan et al., 2019; Lee et al., 2020; Guo et al., 2021). It is hypothesized that metformin acts on T cells and can regulate their function through metabolic signaling pathways such as mechanistic targets of rapamycin (mTOR) and AMPK (Nyambuya et al., 2020). In fact, in young mice metformin promotes the formation of memory CD8^+^ T cells though the activation of AMPK and enhancement of fatty acid oxidation (Pearce et al., 2009; Zhang et al., 2020). Thus, metformin has direct immunometabolic effects that alters functionality. Additionally, metformin was found to attenuate reactive oxygen species via FOXO3 activation, a transcription factor involved in inflammation, cell metabolism, and longevity in human peripheral blood mononuclear cells (PBMCs), composed of T cells, B cells, NK cells, and monocytes (Hartwig et al., 2021). Metformin was also able to reduce reactive oxygen species and reactive nitrogen species levels in these cells in a dose dependent manner (Hartwig et al., 2021). Further, metformin can improve age-related changes in B cells. It was determined that metformin used *in vitro* to stimulate B cells from recently diagnosed type 2 diabetic patients was able to reduce B cell-intrinsic inflammation and increase antibody responses (Diaz et al., 2017). Additional research from this group demonstrated metformin can decrease the frequencies of pro-inflammatory B cell subsets and intrinsic inflammation of peripheral B cells from type two diabetic patients (Frasca et al., 2021).

Thus, metformin has the capability to impact immune responses through its modulation of inflammation, the microenvironment, and via metabolic and non-metabolic action on immune cells themselves. These findings highlight the ability of metformin to modulate immune cell function, specifically in T cells, macrophages, and B cells which are essential for the controlling responses to infection and generating long-term immunological memory. Overall, increasing research demonstrates a potential case for metformin in increasing immunological resilience in older adults (Justice et al., 2021). Flu and SARS-CoV-2 are leading killers among older adults due to immune declines with aging, and metformin has potential to impact health outcomes when older adults contract one of these viral illnesses. Clinical trials have demonstrated the potential thus far and additional clinical trials are now underway to examine the ability of metformin to improve responses to vaccination or infection with flu and/or SARS-CoV2.

## Clinical trials with metformin during acute SARS-CoV-2 or influenza infection

With the emergence of the COVID-19 pandemic, numerous clinical trials are now looking to reduce disease severity and mortality after pathogen exposure. Indeed, multiple clinical investigations have already explored FDA approved drugs to reduce the huge burden of COVID-19 in at-risk individuals. Metformin was one of the leading treatments tested with many hypotheses focusing on reducing the cytokine storm and excessive inflammation during SARS-CoV-2 infection. A systematic review and meta-analysis of observational data in COVID-19 patients highlighted an association between metformin use and a significantly reduced risk of hospitalization and mortality (Li et al., 2021). In addition, two retrospective studies from the National COVID Cohort Collaborative (N3C) database showed potential benefits of metformin treatment in patients with COVID-19. One study determined that metformin use was beneficial in preventing severe COVID-19 outcomes in subjects with prediabetes or polycystic ovary syndrome (PCOS, a condition that is commonly treated with metformin off-label) compared to control-matched participants (Chan et al., 2022). Another retrospective study evaluated the associations between metformin use and COVID-19 outcomes in diabetics compared to diabetics on other therapeutically equivalent diabetes monotherapies (Bramante et al., 2022a). This study showed that metformin use in diabetics was associated with less severe COVID-19 compared with sulfonylurea use but not dipeptidyl-peptidase-4-inhibitors, two other common diabetes medications (Bramante et al., 2022a). More specifically, metformin use was associated with a lower risk of ventilation and mortality (Bramante et al., 2022a). Further studies also determined that diabetic metformin users who were hospitalized with COVID-19 had a shortened hospital stay duration, a lower rate of intubation, and decreased inflammatory markers compared to diabetics on other oral hypoglycemic medications (Usman et al., 2022). These retrospective studies highlight the association of metformin use and improved COVID-19 outcomes.

While retrospective studies provide associations, as the COVID-19 pandemic progressed, prospective studies were designed to determine the potential benefits of metformin use in COVID-19 patients irrespective of diabetes status. The COVID-OUT [Early Outpatient Treatment for SARS-CoV-2 Infection (COVID-19)] trial was a blinded, placebo-controlled randomized trial of metformin, ivermectin, and fluvoxamine for the treatment of COVID-19 (Bramante et al., 2022b). Eligibility criteria in this trial focused on individuals who were at high risk for severe infection and included adults between the ages of 30 and 85, a body mass index associated with overweight or obesity, proof of SARS-CoV-2 infection within the past 3 days, and onset of symptoms within 7 days before drug randomization (Bramante et al., 2022b). None of the three drugs had a significant effect on the primary outcome of this trial, hypoxemia. However, secondary analyses determined that metformin had benefits for many of the secondary end points of the trial (Bramante et al., 2022b). More specifically, metformin lowered the odds of emergency department visits, hospitalizations, and death due to COVID-19 (Bramante et al., 2022b). A follow up study from the COVID-OUT clinical trial determined that there was a 42% relative decrease in the incidence of Long COVID in the metformin group compared to the placebo group (Bramante et al., 2022c). Additionally in a recent pre-print, viral load was reduced 3.6-fold with metformin relative to placebo while there was no virological effect for ivermectin or fluvoxamine (Bramante et al., 2023). These findings from the COVID-OUT trial are particularly promising since the subjects included diverse group of adults 30–85 years, and not only those with prediabetes or diabetes. These findings suggest that metformin treatment can be beneficial in dealing with acute COVID-19 infections in all moderate-risk individuals, as well as attenuate Long COVID symptoms.

These results were replicated in another trial of hospitalized patients with severe acute respiratory syndrome secondary to SARS-CoV-2 and diagnosis of T2D. The results of this randomized, double-blind, phase IIb clinical trial showed that patients treated with metformin had a reduction in SARS-CoV-2 viral load which reached an undetectable level in an average of 3.3 days as compared to the control group which took an average of 5.6 days (Ventura-Lopez et al., 2022). Metformin treatment also reduced supplementary oxygen requirements compared to the control group (Ventura-Lopez et al., 2022). Complimentary *in-vitro* studies provided a potential mechanism via a direct reduction of SARS-CoV-2 viral replication in Vero E6 cells with metformin treatment, suggesting metformin has direct anti-viral action against SARS-CoV-2 (Ventura-Lopez et al., 2022). Although more research still needs to be completed, early clinical trials with metformin demonstrate the potential benefits of this drug in patients battling COVID-19 infection.

Interestingly, metformin was originally investigated as an anti-influenza drug in the early 1940s and showed some promise in improving flu symptoms coupled with reducing blood glucose levels (Garcia, 1950; Bailey, 2017). While it was not directly pursued as an anti-influenza drug, metformin showed promise in a variety of infections. Further retrospective studies suggest that metformin has protective benefits during flu infection as well. For example, obese patients with a history of metformin treatment have been shown to have a lower rate of influenza mortality (Cum et al., 2022). Another study demonstrated that in diabetics, metformin treatment reduced overall risk for hospitalization due to infections compared to other oral hypoglycemics such as sulfonylureas (Mor et al., 2016). Thus, retrospective studies suggest metformin has utility in flu infections, however prospective randomized trials have yet to be completed.

Overall, these data suggest that metformin treatment has great potential to improve health outcomes during acute infection with either flu or SARS-CoV-2. Since both of these pathogens result in increased mortality and disability post-infection in older populations, the ability to repurpose already FDA approved drugs such as metformin to combat these viral infections would be extremely beneficial for older adults. Currently the average clinical development time for new innovative drugs is 9.1 years (Brown et al., 2022). Although a variety of factors can impact this, repurposing already FDA approved drugs can save significant amounts of time and money, further enforcing its potential as a therapeutic in the face of pathogens such as SARS-CoV-2 and flu in at-risk populations. Future studies will better define mechanisms and determine if certain individuals may benefit greater than others from metformin treatment during acute infections.

## Clinical trials with metformin and SARS-CoV-2 or influenza vaccination

Importantly, both flu and COVID-19 have available vaccines that significantly reduce the likelihood of infection and severity of infection. While seasonal flu vaccine is reformulated every year due to mutations to the flu virus, the COVID-19 vaccination has only been reformulated twice thus far since the pandemic started and the vaccine became available. For both of these infectious diseases, vaccination is the best way to prevent severe disease and mortality and is highly recommended for at-risk populations (Smetana et al., 2018; Liang et al., 2022). The seasonal flu vaccine has specific formulations for older adults that improves overall protection compared to the standard flu vaccine (DiazGranados et al., 2014; Smetana et al., 2018). Further, the mRNA-based COVID-19 vaccine seems to have better efficacy in older adults compared to more traditional vaccine platforms (Anderso et al., 2020; Polack et al., 2020; Baden et al., 2021). Nonetheless, older adults still have the highest burden of infection for both flu and COVID-19 likely due to the age-related immune dysregulation discussed above. Older adults have impaired T and B cell responses that lead to poor responses to vaccination (Nikolich-Zugich, 2018; Gustafson et al., 2020), including reduced vaccine-induced antibody quantity and quality, reduced cell-mediated responses, and overall impaired vaccine-induced protection. Thus, therapeutics that could improve vaccine efficacy in older adults would be valuable to prevent flu and COVID-19 infections and reduce severity of disease.

While the previous section noted metformin’s ability to reduce excessive inflammation and improve outcomes during acute infection responses, metformin may also have utility in improving immunological resilience overall and specific immunological memory responses. Preclinical trials in young mice determined that metformin can improve CD8 T cell memory formation (Pearce et al., 2009). Retrospective studies suggest metformin can improve vaccine-induced protection in humans as well. Diabetics on metformin prior to flu vaccination had a lower risk of severe flu infection and associated complications compared to diabetics not on metformin (Yen et al., 2022). More specifically, the risk of hospitalization for influenza, pneumonia, cardiovascular disease, invasive mechanical ventilation, death due to cardiovascular diseases, and all-cause mortality in older adults with T2D was decreased after contracting flu when participants were taking metformin prior to vaccination (Yen et al., 2022). Additionally, a longer cumulative duration of metformin use was associated with lower risks of the outcomes listed above compared with participants that were not using metformin (Yen et al., 2022). This research highlights that metformin treatment during vaccination can considerably improve outcomes after influenza infection in older adults with diabetes. However, since previous work shows the benefits of metformin on acute flu infection responses, it is difficult to determine the impact of metformin benefits is solely due to improved vaccine-induced protection. Interestingly, another retrospective study provided additional clarification in terms of if metformin can improve flu vaccine-induced antibody production. Metformin treated diabetics had increased serum flu antibodies compared with recently diagnosed diabetic patients not on anti-diabetic drugs (Diaz et al., 2017). Further *in vitro* studies confirmed metformin improved B cell specific responses in older diabetics as well as noted above (Diaz et al., 2017). Thus, these studies support that metformin treatment can improve humoral responses to vaccination in patients with T2D.

Prospective studies have also confirmed the benefits of metformin on flu vaccine responses in old diabetic patients on metformin compared to old diabetics not on metformin. More specifically, in a randomized control trial, metformin significantly increased serum flu vaccine-specific antibodies in old individuals with T2D (Frasca et al., 2021). Importantly, this study showed that metformin increased antibody responses to flu vaccination in patients with T2D to similar levels as both healthy older adults and healthy young adults (Frasca et al., 2021). Mechanistically, metformin decreased B cell intrinsic inflammation, a known factor that is negatively associated with protective responses to vaccination, in T2D (Frasca et al., 2021). Ways to improve B cell function and increase antibody-mediated protection after vaccination is critical when combatting infectious disease. Therefore, metformin’s ability to improve B cell function and antibody responses represents a novel way to increase vaccine-induced protection in diabetic patients.

While previous research demonstrates clear benefits of metformin on vaccination responses in diabetics, recent research from our lab has begun to investigate the potential of metformin to improve flu vaccination responses in healthy older adults (≥65 years old). In a double-blinded, placebo-controlled pilot trial, non-diabetic and non-prediabetic older adults were randomized to either metformin or placebo treatment for a total of 20 weeks with flu vaccination occurring following 10 weeks of treatment. The aim of this study was to interrogate the ability of metformin to improve flu vaccine responses and overall immunological resilience in a population of healthy older adults. We aimed to explore the utility of metformin outside of the known benefits in diabetics and prediabetics; thus, our trial excluded any individuals with prediabetes or diabetes. Although serum antibody responses were not increased by metformin use, metformin treatment resulted in an increase in circulating T follicular helper cells (cTfh) post-vaccination (Martin et al., 2023). This suggests that metformin modulates flu vaccine responses in healthy older adults, particularly favoring cTfh cell-mediated responses. The significance of this finding, however, requires further research in a larger cohort to determine if increased cTfh would lead to meaningful changes in antibody quantity and/or quality. Further, we showed that 20 weeks of metformin treatment decreased certain markers of T cell exhaustion in this population of healthy older adults (Martin et al., 2023). Our pilot study suggests that metformin improves some aspects of T cell responses in healthy non-diabetic older adults. More research is needed to confirm these findings and expand to larger populations to determine if metformin can improve flu vaccine efficacy overall in older non-diabetic adults.

While COVID-19 vaccines tend to have high efficacy overall, retrospective studies have also investigated the impact of metformin on vaccine responses. Metformin use before COVID-19 vaccination did not reduce the incidence of COVID-19. However, in a retrospective cohort study, metformin use before COVID-19 vaccination reduced the risk of hospitalization, complications, and mortality in patients of different ages, sexes, races, those with diabetic complications, and with or without insulin use (Yen et al., 2023). It is unclear, however, if benefits from metformin in this study are due to improved vaccination responses or improved immune responses during acute infection. Although further research is needed, the ability to increase the protective effects of vaccines with metformin could be extremely beneficial in older populations.

## Conclusion

**FIGURE 1:**
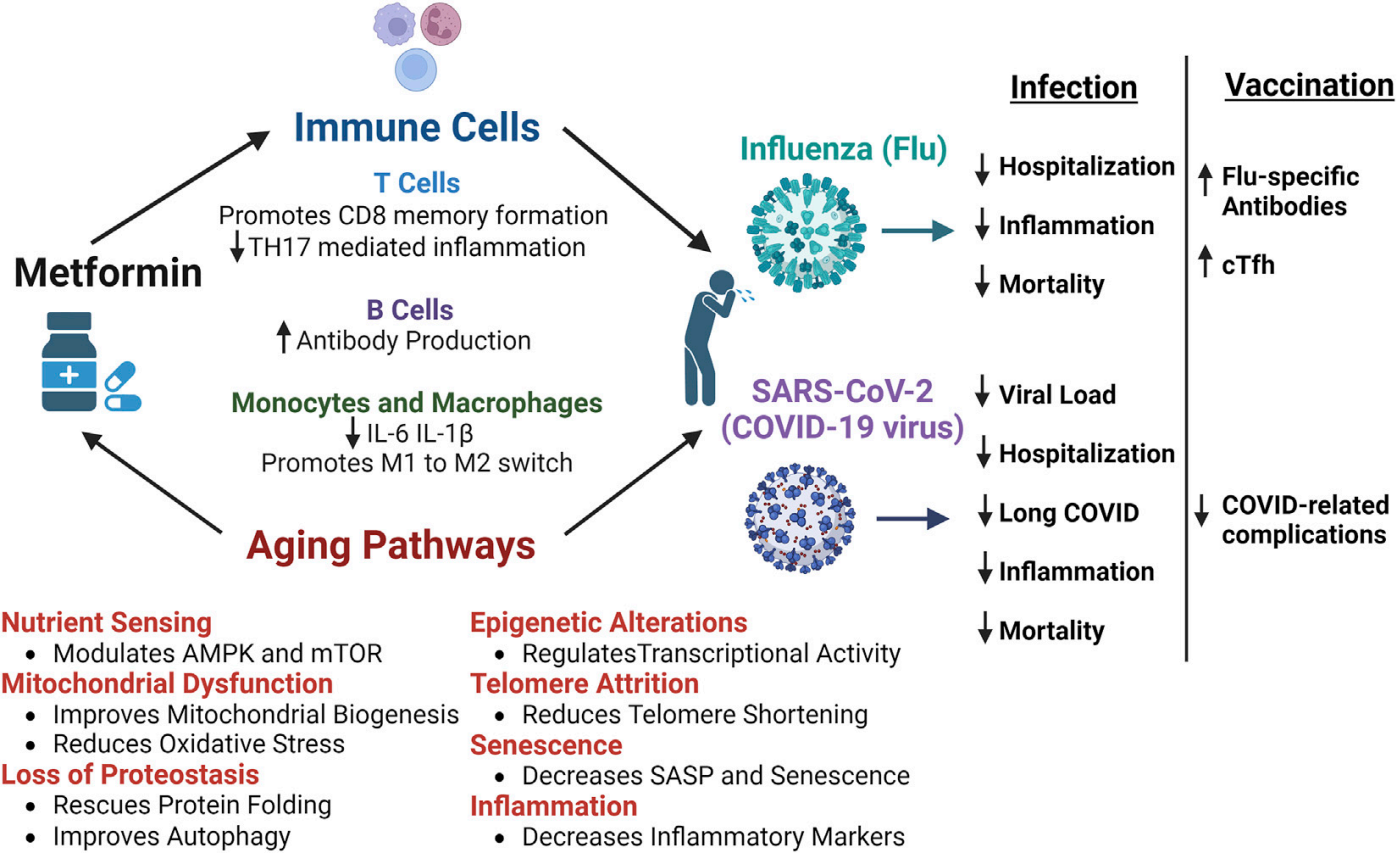
Benefits of Metformin and Potential Mechanism of Improved Responses to Influenza and SARS-CoV-2 infections. The multiple mechanisms by which metformin can improves outcomes after infection with influenza (flu) or SARS-CoV-2 (COVID-19 virus). Metformin can directly impact T cells, B cells, monocytes, and macrophages to control inflammatory responses and promote immune-mediated protection. Metformin also acts on numerous aging pathways, such as nutrient sensing, mitochondrial dysfunction, loss of proteostasis, epigenetic alterations, telomere attrition, senescence, and inflammation, that are dysregulated with aging and lead to decreased resilience in older adults. Retrospective and prospective studies have shown that metformin improves certain outcomes after infection with flu or SARS-CoV-2. More specifically, metformin leads to reduced hospitalization and mortality, decreased SARS-CoV-2 viral load, reduced incidence of long-COVID, and better control of inflammatory responses to infection. Additionally, after flu vaccination, metformin was shown to increase flu specific antibodies in diabetics and circulating T follicular helper cells (cTfh) in non-diabetic older adults. Further, individuals vaccinated against COVID while taking metformin had reduced complications post-infection. Thus, the cumulative benefits of metformin have the potential to improve infection outcomes and overall immunological resilience in older adults. Figure created with Biorender.com.

In conclusion, metformin is an attractive candidate to improve immune defenses and resilience in older adults facing viral challenge as outlined in Figure 1. Metformin is already a candidate anti-aging drug, but its benefits have potential to span beyond this and improve specific immune responses. Metformin can target multiple aging hallmarks such as deregulated nutrient sensing, mitochondrial dysfunction, inflammation, cellular senescence, loss of proteostasis, epigenetic alterations, and telomere attrition. Moreover, metformin can directly impact immune cell subsets such as T cells, B cells, monocytes, and macrophages which are known to become impaired with aging. Both retrospective and prospective studies have demonstrated metformin’s efficacy in improving outcomes after SARS-CoV-2 or flu infections. Additionally, further evidence from clinical trials has suggested that metformin treatment can improve vaccination responses.

In the face of viral pathogens such as influenza and SARS-CoV-2, strategies to improve outcomes after infection or improve vaccine-induced protection are invaluable. This is especially important for older adults who are at increased risk of death or worsened outcomes post-infection. Although clinical trials investigating the ability of metformin to improve vaccination responses or outcomes post-infection are currently under way with promising results, further research with larger cohorts is needed. Additional trials and experiments are necessary to determine the mechanism by which metformin is inducing protection and modulating immune responses. Nonetheless, early results are promising and highlight the ability to repurpose FDA approved therapeutics to combat infectious disease to improve immunological resilience with aging.
